# Production of Polyhydroxybutyrate (PHB) and Factors Impacting Its Chemical and Mechanical Characteristics

**DOI:** 10.3390/polym12122908

**Published:** 2020-12-04

**Authors:** Blaithín McAdam, Margaret Brennan Fournet, Paul McDonald, Marija Mojicevic

**Affiliations:** Athlone Institute of Technology, Dublin Road, N37HD68 Athlone, Ireland; a00277255@student.ait.ie (B.M.); mfournet@ait.ie (M.B.F.); pmcdonald@ait.ie (P.M.)

**Keywords:** biosynthetic polymers, biodegradable, biosynthesis, bacterial fermentation, polyhydroxyalkanoates, PHB

## Abstract

Plastic pollution is fueling the grave environmental threats currently facing humans, the animal kingdom, and the planet. The pursuit of renewable resourced biodegradable materials commenced in the 1970s with the need for carbon neutral fully sustainable products driving important progress in recent years. The development of bioplastic materials is highlighted as imperative to the solutions to our global environment challenges and to the restoration of the wellbeing of our planet. Bio-based plastics are becoming increasingly sustainable and are expected to substitute fossil-based plastics. Bioplastics currently include both, nondegradable and biodegradable compositions, depending on factors including the origins of production and post-use management and conditions. Among the most promising materials being developed and evaluated is polyhydroxybutyrate (PHB), a microbial bioprocessed polyester belonging to the polyhydroxyalkanoate (PHA) family. This biocompatible and non-toxic polymer is biosynthesized and accumulated by a number of specialized bacterial strains. The favorable mechanical properties and amenability to biodegradation when exposed to certain active biological environments, earmark PHB as a high potential replacement for petrochemical based polymers such as ubiquitous high density polyethylene (HDPE). To date, high production costs, minimal yields, production technology complexities, and difficulties relating to downstream processing are limiting factors for its progression and expansion in the marketplace. This review examines the chemical, mechanical, thermal, and crystalline characteristics of PHB, as well as various fermentation processing factors which influence the properties of PHB materials.

## 1. Introduction

Polymer material usage continues to expand relentlessly, with the global plastic demand almost doubling since 2000 and outpacing all other bulk materials, such as steel, aluminium, or cement. The broader plastics and related commodity production from petrochemical feed stocks drives up to 12% of global oil demands. Non-renewable resource depletion and subsequent greenhouse gas emissions combined with the absence of adequate technologies for post-use circularity and inappropriate disposal of these non-biodegradable materials is leading directly to our perilous environmental situation. Alternative biodegradable and environmentally sustainable biopolymers such as Polyhydroxyalkanoates polyhydroxyalkanoate (PHA) and its homopolymer, polyhydroxybutyrate (PHB), hold the potential to replace plastics linear use and dispose practices with a fully circular life-cycle for plastics.

Mechanically, PHB materials are typically stiff and brittle in nature, with low thermal stability and a high degree of crystallinity. Many PHB plastics have properties that are similar to the petroleum polymers polypropylene (PP) and polyethylene (PE). Feedstocks for PHB biopolymer production include renewable and sustainable sources such as food waste. These factors, combined with its biocompatibility and predisposition to biodegradation on exposure to designated active biological environments make PHB a leading candidate as an alternative to synthetic polymers such as PP and PE. Since its discovery in the 1920s [1], PHB has been extensively studied by microbiologists, as carbon and energy storage products within metabolic pathways of various bacterial strains. It has also been studied by polymer scientists and engineers as the material that exhibits unique characteristics, a number of which are superior to corresponding synthetic polymers. However, several limitations currently exist when PHB is produced at industrial scale capacities. The high costs of production, low yield, susceptibility to degradation, technology complexities including extraction difficulties, are among the challenges facing PHB production [2]. In order to reduce the costs of production and achieve economic viability in competition with the current low petroleum plastics manufacturing costs, a series of new emerging bacterial fermentation processes are being evaluated [3], which have the potentially to accentuate the kinetics of microbial growth and PHB accumulation to the levels necessary for profitable process engineering and production serving the plastics value chain stakeholders.

When PHB materials are produced through different bacterial fermentation processes, the accumulation of the material is variable and is widely documented in a number of reports. In striving to determine if the use of different fermentation processes such as discontinuous methods (batch, fed-batch) versus continuous methods (two-stage chemostat) have an impact on the chemical structures and properties of the resulting polymer material, side-by-side studies of the different processes, and its impacts on the material were considered. There are numerous studies reported in literature on the production of PHB through continuous and discontinuous fermentation processes, however they mainly focus on the accumulation of the material, the yield, and purity. In this review, the objective was to determine if the adoption of various fermentation process’ factors has an impact on the chemical and mechanical properties of the resulting PHB material.

## 2. Polyhydroxyalkanoates (PHAs)

**Figure 1:**
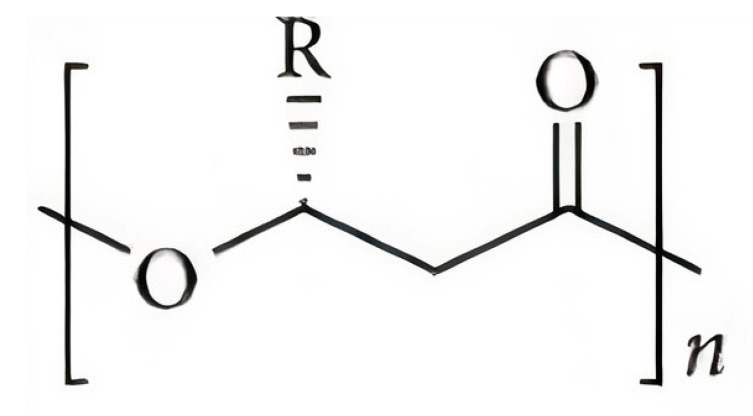
Typical chemical structure of polyhydroxyalkanoate (PHA) molecules [5].

Polyhydroxyalkanoates (PHAs) are a family of microbial polyesters which comprise of a large family of thermoplastic polymers. They are produced by a variety of prokaryotic microorganisms under unbalanced nutrition conditions as carbon and energy storage materials [4]. Materials within the PHA family can differ significantly in their chemical structure. The general structure of the repeating unit of PHA is represent here in Figure 1, where *n* can be greater than zero and provides the number of repeating units in the polymer chain and *R* represents a functional group in the structure, varying on the type of PHA.

The monomer units of PHA can be classified into two groups based on the number of carbon atoms: short-chain and medium-chain length PHAs. The length of the monomer unit plays a critical role in the resulting polymer properties and therefore, prior knowledge of the monomer chemical structure is required to target specific properties of the material for a number of different applications. Short-chain length PHAs (scl-PHA) comprises of 3–5 carbon atoms. Some examples of the monomeric units for scl-PHAs include: 3-hydroxybutyrate (3HB), 4-hydroxybutyrate (4HB), or 3-hydroxyvalerate (3HV). The scl-PHA materials have thermoplastic properties similar to that of polypropylene [6]. As PHB contains a methyl group in its chemical structure, it is the most prominent representative of the scl-PHA group. However, mcl-PHB materials are also possible, with studies reporting that *Pseudomonas* isolates LDC-5 and LDC-25 are capable of producing mcl-PHB material [7]. mcl-PHA consist of 6–14 carbon atoms, which could include some of the following monomeric units: 3-hydroxyhexanoate (HHx), 3-hydroxydecanoate (HD), or even longer-chain comonomer units [8]. As mcl-PHAs have a higher carbon chain length, they show a reduced crystallinity and increased flexibility that resemble elastomers and latex-like properties. They also have a low glass transition temperature and lower molecular mass when compared to scl-PHAs [9]. A recent study revealed that the presence of over 30% long chain comonomer units in mcl-PHAs could increase the melting temperature and the degree of crystallinity, resulting in a significant change in mcl-PHA mechanical properties and also differentiating them from the typical mcl-PHA material [8]. There is also a difference in the purity levels between the two classes. An example is in the case of solvent extraction using acetone, where the scl-PHA purity and recovery is much higher than that for mcl-PHA materials. The purity of scl-PHA is reported to be around 98.4% and recovery of 96.7%, while the purity of mcl-PHA ranges between 80% and 90% and the recovery rates range between 60% and 80% [10]. In order to produce an overall superior PHB material, a number of studies have been conducted aiming to improve the mechanical behavior and reduce the high costs associated with the material production. Some of these approaches are based on the use of blends, the addition of functional groups, copolymer developments and chemical modifications of the material [11,12,13,14,15]. Functionalization of PHA copolymers showed important improvement in the properties and appears to be a very good approach in increasing the applications of PHA materials. A recent example is shown by Bhatia et al., where poly-(3-hydroxybutyrate-co-3-hydroxyvalerate) (PHBV), functionalized with ascorbic acid, resulted in a lower degree of crystallinity (9.96%), higher thermal degradation temperature (294.97 °C) and hydrophilicity (showing contact angle of 68°) and 1.6 fold increase in biodegradability when compared to the un-functionalized PHBV copolymer [15].

## 3. Polyhydroxybutyrate (PHB)

**Figure 2:**
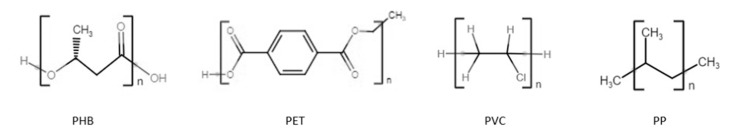
Chemical structures of P3(HB) in comparison to commonly used petroleum-based polymers (polyethylene terephthalate (PET), polyvinylchloride (PVC), PP).

Poly(3-hydroxybutyrate) (P3(HB)) was the first isolated and characterized amongst PHAs. P3HB is highly crystalline due to its linear chain structure, containing both amorphous and crystalline phases. It can be found as a virgin polymer or as part of copolymers and blends. It is generated as a carbon reserve in a wide variety of producing bacterial strains and is produced industrially through bacterial fermentation. P3(HB) also has a number of advantages over synthetic polymers for the production of certain packaging applications including: P3(HB) barrier permeability is superior to both polyethylene (PE) and polypropylene (PP), and they are also found to be more rigid and less flexible than PP (Figure 2). Besides that, PHB exhibits good barrier properties in comparison to polyethylene terephthalate (PET) and polyvinylchloride (PVC) [16]. Another main characteristic of P3(HB) material is its biodegradability, occurring within a reasonable timescale when the material is in contact with degrading microorganisms in biologically active environments such as soils, fresh water, and aerobic and anaerobic composting, designating them as sought-after eco-friendly alternative for synthetic polymers [17].

As a member of the PHA family, PHB is characterized by having a methyl functional group (CH_3_) and an ester linkage group (−COOR), it is these functional groups that are responsible for the materials thermoplastic, hydrophobic, high crystallinity, and brittle characteristics. The thermal properties of semi crystalline materials, such as PHB and its derivatives, typically include two main temperatures: a glass transition temperature (Tg) for their amorphous phase and a melting temperature (Tm) for the crystalline phase [18]. There is also the degradation temperature (Td), the temperature at which the material can start to degenerate. There are a number of different analytical methods that can be used for measuring the Tm, Tg, and Td such as the Differential Scanning Calorimetry (DSC), X-Ray Diffraction (XRD). The degree of crystallinity can also be calculated using these analytical methods. FTIR analysis is a widely used analytical technique, providing information about the molecular structure of samples and the determination of the purification level. In recent years it has also been used for the investigation of the crystallization of PHA materials [19]. In Table 1, representative mechanical properties of P3(HB) are displayed [18,20,21,22,23] in comparison to PP [24], PET [25], PE (LDPE—low density PE [26] and HDPE—high density PE [27]) and PLA (PLLA and PDLLA) [25,28].

Crystallinity can be referred to as the degree of structural order and regularity in molecular arrangements of polymer materials [29] and is one of the most important characteristics of a polymer as it is dominant in determining the mechanical properties of the plastic material [30]. For P3(HB), Vroman et al. reports a crystallinity value above 50%, while a value above 60% is reported by Sharma and Ray [31,32]. There are a number of studies reporting ranges of crystallinity, such as a report by Jendrossek and Handrick stating that the typical degree of crystallinity of P3(HB) is 50% to 60% [33], while Kansiz et al. provides a range of crystallinity of 60% to 70% for P3(HB) materials [19] and Santos et al. acknowledged a crystallinity range of P3(HB) being between 60% to 80% [34]. In general, the higher the degree of crystallization results in a stiffer and stronger, yet more brittle material. The degree of crystallinity can also have an influence on the tacticity of the polymer, hardness, modulus, density, transparency, and the nature of cold drawing or ductile flows [35].

One challenge associated with PHB materials is their narrow processability window, specifically the narrow difference between the Tm and Td, resulting in this material being susceptible to thermal degradation at temperatures in the region of the melting point [31,36]. A number of different approaches have been evaluated to widen the processability of PHAs. For example, the Tm of PHA copolymers can be adjusted by changing the ratios of the monomers used for polymerization. When the percentage of 3 HV added to produce PHA copolymers is increased from 0 to 25 mol% [37], the Tm and the degree of crystallinity can be decreased without significantly impacting the Tg and Td, consequently widening the process window and improving the melt processability [6]. The incorporation of additional units of 3 HV has also been shown to improve the impact strength, however this is also accompanied by a reduction in tensile strength and modulus [38]. It has also been reported that the Tm of poly-(3-hydroxybutyrate-co-4-hydroxybutyrate) decreases from 176 °C to 54 °C when the 4HB content increases from 0 to 38 mol%, however it appears that a plateau is reached with a further increase of 4HB when the system passes the pseudoeutectic point [39]. In comparison to P3(HB), P4(HB) is a relatively new material. It is synthesized either through the condensation reaction of 4-hydroxybutyric acid (4HB) or through the ring-opening polymerization (ROP) of the γ-lactone [40]. These are generally non-toxic, biocompatible, optically active, presents relatively good barrier to the permeability of water and gases, are stable when placed under UV. Radiation conditions present with a yellow hue when the purity is high and can be processed by extrusion, injection molding, blowing and thermoforming [34]. As they are generally non-toxic and biocompatible, they can be used in the biomedical field within medical devices, as they will not be rejected by bio-environments in which they are implanted or placed in.

Among various reports on P3(HB) materials, different thermal values have been reported, for example some authors suggest that the Tg is approximately 5 °C to 6 °C [20,41]. However, values ranging from 0 °C to 4 °C, and −15 °C to 9 °C have also been reported for P3(HB) and its copolymers [32,42]. Likewise, a variation in the Tm of PHB materials can be found in the literature, with a range of 160 °C to 180 °C covering most of the temperatures reported [43].

In general, polymers with a low degree of crystallinity have been found to demonstrate a wider processing window, while polymers with a higher degree of crystallinity usually showed a narrow window, due to a sharper melting range [44]. The thermal properties and crystallinity of the polymers are important characteristics to measure and characterize as they can provide a wealth of information, allowing material researchers and scientists to predict and control the mechanical properties and processability of the PHB materials produced. In further text, P3(HB) will be referred to as PHB.

### 3.1. PHB Synthesis

PHB is produced in the cells of microorganisms [45], as product of microbial secondary metabolism, usually in conditions when the cells are subjected to nutrient stress or in an unfavorable environment such as carbon-excessive with limited nutrients [46], which is possible in both gram-positive and gram-negative bacteria. The accumulation of the material is a natural technique used by microorganisms to store carbon and energy when essential nutrient supplies are imbalanced or depleted [47]. It is important to note that there are a number of different species of bacteria which have been known to accumulate materials, such as PHBs as intercellular granules, with reports stating that this number may exceed 75 different genera [48].

**Figure 3:**
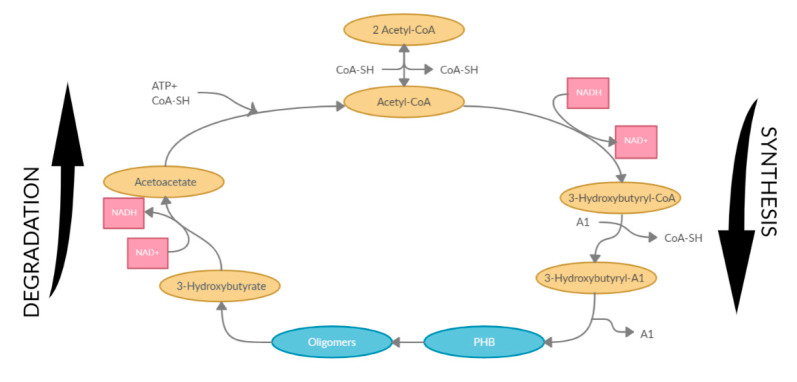
Polyhydroxybutyrate (PHB) synthesis and degradation process.

There are several different approaches for the extraction and recovery of PHB materials and its derivatives from bacterial cells. This topic generates a great amount of interest as different forms of the PHB materials can be synthesized, depending on the microorganism used and, on the approach selected for obtaining the material. For example, Sudesh et al. details that an isotactic PHB with little to no stereoregularity is obtained when a bacterial process is utilized, all in the R-configuration due to the stereospecificity of the polymerizing enzyme, PHA synthase. In very rare cases, a small percentage of the S-configuration can be detected, while the syndiotactic PHB with stereoregularity can be achieved through chemical synthesis [31,49]. There are three main routes of synthesizing PHB materials outlined by Vroman et al., the first approach being through ring opening polymerization (ROP) of β-butyrolactone (BL) [31]. Another approach is through the use of natural/transgenic plants. The biosynthesis of PHAs in transgenic plant cells is possible because of the general availability of acetyl-CoA, the primary substrate in PHA biosynthesis, as is the case for example with Linum *usitatissimum L.*, also referred to as flax [50]. This plant has been used by humans dating back to ancient times, but advancements in technology relating to transgenesis allowed the prospect of modifying flax plants and also allowed for higher biomass growth, with about a 20% yield increase in comparison to control cultures. It was also observed that the cellulose in the plant cell walls of transgenic callus was structurally different, with little organization when compared to the control callus, resulting in a lower degree of crystallinity [51]. With an increasing demand on biodegradable resources in the future, use of transgenic plants is an approach that is being further developed to obtain highly efficient bioprocesses, and is an area showing immense potential. The third approach of obtaining PHB materials is through bacterial fermentation. When under optimal fermentation conditions, it is possible that more than 90% of the cells dry weight may be comprised of PHA materials [52]. This third approach is the most commonly used in the synthesis of PHB (Figure 3). PHB synthesis relies on a central carbon metabolite from acetyl-CoA through a sequence of three enzymatic reactions:

1. The reversible condensation of two acetyl-CoA moieties forming acetoacetyl-CoA, catalyzed by β-ketothiolase (PhaA);
2. Acetoacetyl-CoA reduction to (R)-3-hydroxybutyryl-CoA by an acetoacetyl-CoA reductase (PhaB);
3. The polymerization of (R)-3-hydroxybutyryl-CoA catalyzed by the enzyme PHB synthase (phbC gene) to produce PHB. The biosynthetic pathway of PHB from acetyl-CoA [5,53].

### 3.2. PHB Fermentation Methods

**Figure 4:**
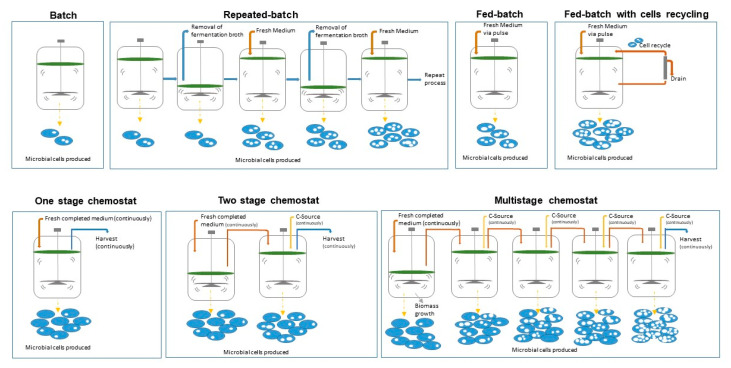
Fermentation processes commonly used for PHA biosynthesis [3].

A number of different fermentation processes can be used to obtain PHBs. These include: discontinuous processes such as batch culture, fed-batch culture, and repeated fed-batch culture [54]; and continuous processes such as continuous fed-batch systems using gaseous substrates, one-stage chemostat process, two-stage chemostat process, and multi-stage chemostat process in continuously stirred tank reactor (CSTR)-bioreactor cascades [3]. It has to be emphasized that fundamental differences exist between the possibilities to run continuous processes for formation of, on the one hand, extracellular products as listed in the prior paragraph, and, on the other hand, for intracellular products such as PHB which are the topic of this review. Figure 4 provides a schematic overview of the fermentation processes mentioned, which also includes an illustration of the microbial cells produced at the end of each process, differences in density, and PHA mass fractions.

The batch culture process is a simple discontinuous process used for the production of PHB materials, however, is one of least productive methods. At the beginning of the fermentation batch, the concentration of the nitrogen and carbon sources is restricted by the physiological preconditions of the production strain [3]. Due to the low productivity associated with batch culture processes, a simple ‘repeated batch’ approach has recently been evaluated to enhance the volumetric productivity. In the study by Gahlawat et al., a drain-and-fill approach of repeated-batch cultivation was adopted for the enhanced production of PHB using the bacterial strain *Azohydromonas australica.* The report emphasized that the volumetric productivity was successfully increased and displayed a major advantage over simple batch processes in the production of PHB materials. This new approach also eliminates the non-productive time required for the cleaning, refilling, and sterilization of the bioreactor between individual batches [55].

In the fed-batch systems, the addition of the precursor substrate is usually feed through pulses when the concentration falls below a set value. However, without a proper feeding strategy this culture would not result in a much higher productivity than observed for batch culture processes [56]. A number of reports have determined optimal feeding rates of carbon and nitrogen sources based on the material being processed. In the case of PHA, one example is when the nitrogen and carbon sources are refed into the process according to the consumption of the biomass up until a PHA-poor biomass is achieved [3]. One of the main challenges associated with fed-batch fermentation is the control over the feeding substrate concentration to allow an ideal range in terms of limiting and inhibiting levels. Therefore, the substrate feeding approach used for the successful production of a high percentage of accumulated PHB is essential [56]. To enhance the fed-batch approach, several feeding strategies have been reported, such as the use of a two feeding-pulse fed-batch strategy, which can successfully obtain the highest volume of PHB and polymer concentration [57], as well as a number of continuously feeding approaches. All in all, discontinuous processes feature limited productivity, mainly due to the time needed for preparation and post-treatment of the bioreactor. In addition, product quality can fluctuate between different batches in terms of molar mass distribution [58].

Even though batch and fed batch processes are well known and established methods for PHB materials production, emerging fermentation processes are being further studied, with continuous feeding considered to be the simplest and the most ideal method for the PHB production when compared with other methods [59]. The continuous fermentation processes are recognized to operate under steady, controlled conditions, where factors that can affect the process such as the pH, nutrient supply, and concentration of the product are kept constant. These approaches are well-known in many industries based on microbial production, such as the beverage industry. The same approach can be used for successful continuous cultivation to gain extracellular products in the conversion of starch to lactic acid by *Lactobacillus amylovorus* [60]. These examples are in clear contrast to the PHB-case; here, a sufficient number of active cells have to be formed under nutritionally balanced conditions in a first stage; in a second stage, these cells have to accumulate the biopolymer as an intracellular product. The constant conditions required to generate high active biomass, however result in only a small fraction of accumulated material [3]. This is due to the fact that PHB production depends on the physiological stress response placed on the microorganisms when essential nutrients are limited or depleted. In order to produce PHB, a cell growth phase occurs first where the bacteria are fed with essential nutrients to the level needed for PHB production and then nutrients are depleted after a certain time to trigger secondary metabolism and encourage polymer biosynthesis [61]; hence, it is impossible to complete PHB production using a continuous one-step process at sufficient productivity when conditions are kept constant. Therefore, continuous two-stage and multi-stage fermentation processes are better suited for this purpose and hence, this is currently the most common method of producing PHA materials [54], allowing for stable processing conditions and higher productivity.

Continuous fermentation processes can facilitate high productivity of PHB materials, including the production from cultures of high specific growth rates, however, the execution of such cultivation in industry for PHB production has been limited due to the continuous processes being prone to microbial contamination and productivity interruptions leading to financial losses [62]. On the other hand, due to higher volumetric productivity in continuous processes (batch cultivation requires large bioreactor facilities to generate the same output per time) continuous production contributes to lower investment costs of smaller operation facilities. The achievement of higher consistency and uniformity of product quality has been demonstrated using continuous processes for PHB production [63]. It is clear that continuous cultivation for PHB biosynthesis is more efficient in terms of productivity and product quality with subsequent increased efficiency of the downstream processing and improved performance properties of PHB based bioplastics. In addition to the type of bioprocess operated, several further important factors including the choice of producing microorganism(s), cultivation medium, carbon sources, and process factors are discussed in terms of their impact on the production and quality of PHB.

### 3.3. Factors Impacting Chemical and Mechanical Characteristics of PHB

#### 3.3.1. PHB Producing Strains

PHB materials can be produced by many different bacterial strains, with reports stating that more than 300 different bacterial strains are known PHB producers [18]. Some examples of the extensively studied strains used to produce PHB are; *Ralstonia eutropha* (also known as *Cupriavidus necator*), *Alcaligenes spp.*, *Azotobacter spp.*, *Bacillus spp.*, *Nocardia spp.*, *Pseudomonas spp.*, and *Rhizobium spp.*, with *Ralstonia eutropha* being the most extensively studied [64,65]. Imperial Chemical Industries (ICI) were the first company to use this bacterial strain to produce PHB polymers under the trade name Biopol [47]. Yet presently, there are only a small number of bacterial strains that have been successfully used for PHB production at the industrial scale [66].

There are numerous published reviews on PHB accumulation, yield and purity from the different bacterial strains [66,67,68], with only a very limited number of studies focusing on the comparison of different bacterial strains on the mechanical properties. In recent reports, it has been stated that the type of bacterial strain used can be associated with defining the final molecular weights of the resulting material [69], and given that the mechanical and physical properties of plastics and PHB materials are strongly dependent on the molecular weight of the polymeric chains, the bacterial strain used will have a critical impact on the mechanical quality of the PHB generated. One study by Domínguez-Díaz et al. obtained PHB material with a range of molecular weights from cultures of *Azotobacter vinelandii* wild type and mutant derivatives strains and subsequently investigated the differences in the thermal mechanical properties and chemical structures. The results of the study showed that the thermal and mechanical properties are dependent on its molecular weight, except above 1400 kDa, where the Tm and degree of crystallinity values decreased, possibly due to the occurrence of molecular entanglements and inadequate polymer processing conditions. As a result, the elastic modulus decreased owing to the occurrence of large amorphous segments in the material [70]. This study also concluded that the physical properties and chemical structure of the PHBs were strain dependent.

Crystallinity appears to be one of the key characteristics where changes can be detected when PHB is produced from different bacterial strains. A report by Pradhan et al. (2018), observed that the degree of crystallinity for PHB synthesized by Bacillus megaterium and C. nector were found to be 44% and 23%, respectively [41]. Yet, the thermal properties of the materials were found to be similar or enhanced compared with commercial PHB [42]. These studies have revealed that the degree of crystallinity (Xc) is one of the most important characteristics to measure, alongside molecular weight and chemical substructure, in determining the differences in the material mechanical properties and quality dependence on the bacterial strain used. Results of mechanical characterization of PHBs derived after cultivation of two different bacterial strains and properties’ values found in literature are displayed in Table 2 [20,43,71].

#### 3.3.2. Effect of Medium Composition

A well-designed production medium is one of the key factors for successful bacterial fermentation and significantly impacts on the mechanical properties of PHB materials [72]. A report by Oliveira et al. investigated the chemical structure, thermal and crystalline properties of PHB samples produced by solid-state fermentation (SSF). Experiments were conducted using a non-supplemented medium and a supplemented medium with 2.5% (*m/m*) sugarcane molasses; whereas commercial SSF produced PHB was provided and tested as a control sample [73]. The degree of crystallinity obtained for the commercial PHB was 53%, which is approximately 1.16-fold higher than the degree of crystallinity obtained for the SSF samples (45% and 46%). In this study, the molar mass or molecular weight of the samples, had provided a strong influence on the mechanical properties of the material were also determined using gel permeation chromatography (GPC). The mechanical performance of processed PHB is also known to depend on the carbon source used and culture conditions [69]. PHB obtained by SSF displayed essentially an identical chemical structure as PHB produced by submerged fermentation, with the exception of the lower degree of crystallinity and molar mass. According to the results, the SSF samples exhibited a lower degree of crystallinity, which could be due to the tendency of higher molar mass polymers to crystallize at a slower rate, leading to smaller crystals and hence, a lower degree of crystallinity [73]. Grigull et al. investigated use of oleic acid in a broth medium during the production of PHB material by *C. necator*, using glucose and fructose as the carbon source. The degree of crystallinity of the control PHB sample was found to be 70% using DSC, and a glass transition temperature of −4 °C, agreeing with the values reported in literature. The degree of crystallinity, Tg and Tm of the polymer samples showed a decreasing trend when the oleic acid was increased in the nutritional supplement in the broth medium which can be seen in Table 3 [74].

#### 3.3.3. Effect of Carbon Source Present in Media

As mentioned previously, a major problem for the extensive production and commercialization of PHBs is the high production costs compared with plastics derived from petrochemicals. Development of efficient bacterial strains, optimization of cultivation parameters and recovery processes are among the strategies now used in order to improve PHB production [75,76]. Literature data suggests that one of the major downfalls of overall PHB production costs is the high cost of the carbon substrates selected as feed stocks. As such, the selection of economical and frugal carbon substrate is a key aspect, in order to facilitate a viable market total cost for the final product. Up to 50% of the costs are dependent on the precursor substrate materials, mainly the carbon source [77] and therefore, intense researcher is underway on investigating the utilization of more cost-effective substrates. For example, Dalsasso et al. used vinasse and sugarcane molasses as substrate for PHB production [78]. Additionally, a new *Methylobacterium* sp. isolate was able to produce 0.55 g/L PHB using methanol as a sole carbon source under two-stage fermentation [79]. Many studies using agricultural and industrial waste materials have shown through statistical optimization that using waste material could lower PHBs production costs [80]. Selected examples of the effect of substrate cost and PHB yield have on the production cost are displayed in Table 4.

The influence of the carbon source on the resultant PHB thermo-mechanical properties were largely undocumented prior to 2020 with some newly completed studies only recently available to further close this research gap. Sanhueza et al. studied the characterization of PHB produced by *Paraburkholderia xenovorans* supplied with different carbon sources; glucose, mannitol, and xylose. The FTIR analysis showed that the same material was produced from the different carbon sources, however a slight difference in the intensity of the peaks was observed, indicating that either a highly ordered crystalline structure or more amorphous structure was achieved. This result is similar to the effects observed when using different bacterial strains which also induces variation in the molecular weight of the accumulated PHB material similar to the dependence on the type of carbon source [82].

PHBs produced from rice bran and glucose-based carbon sources were also recently compared, revealing that the PHB produced from rice bran carbon sources showed comparable chemical structures to commercial PHB materials, with higher thermal stability and a lower melting temperature than glucose carbon sources in the production of PHB materials [83]. Another study evaluated the influence of organic carbon sources on the formation of the enzymes of autotrophic metabolism. The resulting materials were characterized, concluding that the organic carbon source affected the properties of the PHB material, with differences in the thermal properties and crystallinity degrees being reported. This study by Garcia-Gonzalez et al. also evaluated the differences between the autotrophic production (utilizing CO_2_ as the carbon source and H_2_ as energy source) and the standard heterotrophic growth (utilizing organic compounds as carbon and energy source). A difference between the two groups was observed, with the autotrophic production resulting in a higher Tm, Tg, a higher degree of crystallinity and a lower Td value compared to PHB produced using the heterotrophic approach [84]. These results indicate the high potential to further optimize the output characteristics of the PHBs and to engineer their thermo-mechanical performances to compete with corresponding PE and PP petroleum based plastics at price competitive rates.

#### 3.3.4. Effects of Agitation and Apparatus

Agitation is an important factor in monitoring and controlling the fermentation of materials. It is reported that the type and strength of agitation applied may have a more significant impact on the resulting material than previously realized, and therefore is an important consideration [85]. The apparatus used is another factor to be considered as it also impacts the resulting material quality. This was demonstrated in a recent study, where the resulting degree of crystallinity of PHB produced by *Alcaligenes latus* ranged from 20% to 26%, depending on the apparatus used during the batch fermentation scale-up. The PHB material produced using the orbital shaker showed a lower degree of crystallinity (20%) when compared to production using a bioreactor (26%), possibly due to slightly better process control which influenced the final quality of the extracted polymer. In this study, a standard PHB sample was tested as a control alongside the PHB extracted by *A. latus*, which was found to have a degree of crystallinity of 48% [86].

PHB produced by *A. latus* was also studied by Yezza et al., using maple sap and sucrose as the carbon source. The results showed that the degree of crystallinity was 52.9% and 55.5%, for the maple sap and sucrose substrates, respectively. However, when further reviewing the report, it comments that the crystallinity values of PHB produced by *A. latus* fermentation on maple sap was completed in fermenter while the sucrose was in shake flask [87]. Therefore, it is unknown whether the difference in crystallinity in this case was due to the difference in carbon source or the difference in the fermentation apparatus used.

#### 3.3.5. Effect of Downstream Process

The impact on the resulting PHB material of downstream processing (the recovery of PHB or extraction method used) was also evaluated. After fermentation, PHB materials are accumulated in the bacterial cells and the first step in its recovery would be the harvesting of the cells, usually by centrifugation. Most of the extraction methods involve the use of solvents, which is a suitable approach for producing materials for medical applications due to their high purity levels [47]. However, the extraction of PHB materials from microorganisms significantly increases the processing costs due to the large quantities of solvents being used and therefore are an important consideration when producing these materials at industrial scales.

Many studies have evaluated the impact of the different solvents for the extraction of PHB materials on their recovery efficiency and purity, as it is known that these are mainly dependent on the extraction method employed to isolate the polymer from bacterial cells. More recently, studies have been completed to evaluate the impacts of the extraction method on the resulting thermal and mechanical properties of the materials. A recent study by Aramvash et al. recovered PHB from *C. necator* through solvent extraction using different solvents, and showed that the enthalpy of fusion of the extracted PHB material by ethylene carbonate and DMSO solvents were 16.8 J/g and 17.04 J/g, respectively. These values are significantly lower than for the control sample of 75.67 J/g, indicating that the extracted PHB material is more elastomeric or rubbery in nature. This study also reported that there was no significant difference in molecular weights of extracted material using examined solvent systems. However, in later studies, it was revealed that the temperature during recovery of the material can have a significant effect on the extracted PHB properties, specifically on the thermal properties and the molecular weight. It was shown that PHB molecular weight strongly differs with temperature and duration of heat treatment during extraction, with the highest values of 1.4 × 10^6^ for butyl-acetate-extracted PHB at 130 °C/30 min and 1.2 × 106 for 90 °C/60 min. This was demonstrated in a study which aimed to examine the effects of temperature and heating incubation time during the recovery of PHB from *C. necator* on the resulting yield, purity, and molecular weight. Limitations were observed when the temperature during extraction was increased above the boiling temperature. At that point, the solvent induces polymer degradation which results in molecular weight decrease [88,89]. In addition to molecular weight, an important parameter that can be affected during downstream processing is the polydispersity index which can determine if the final product can be used for specific applications. Fiorese et al. have shown that more homogenous PHB can be obtained using 1,2-propylene carbonate (lower polidispersity values) in comparison to the material extracted with chloroform [90].

#### 3.3.6. Aging of PHB Materials

Soon after the PHB materials are produced, they can undergo slow changes in their amorphous and crystalline properties, resulting in the hardening or weakening of the material. This process is more commonly referred to as the aging of PHB and its nature derives from two phenomena: the secondary crystallization and the physical aging [34].

After the material is processed, the viscous PHB starts to cool down slowly, with the initiation of crystallization occurring as the temperature decreases. This is said to occur in three main steps; the first where cooling occurs without any crystallization, the second part is where the crystallization starts to occur in high rates, and finally, the last stage is where the crystallization succeeds in low rates, reaching secondary crystallization. This is a process where the molecular chains could possibly form imperfect ordered structures over time, even at room temperature, consequently, increasing in the degree of crystallinity as the material ages [91]. An example of this can be observed in a study by Lopera-Valle et al. The crystallinity of a sample of PHB was measured after one day and again after seven days. The results showed that after seven days of aging, the crystallinity of the PHB sample increased from the initial 20.5% to 28.2%. This change in the material could impact its overall mechanical properties, since the degree of crystallinity increases, it will influence the impact resistance, the young’s modulus and elongation at break [92,93]. In another study by Srubar et al., samples of PHB were isothermally conditioned at 15 °C in a desiccated environment for 168 days in order to investigate the effects of aging at storage temperatures slightly above the Tg of the material. The characterization of the material after the 168 days showed significant variation in the mechanical properties, with evidence of an increase in the tensile modulus and decrease in elongation to break [41,42]. This was also accompanied by a small change in the tensile strength.

### 3.4. Property Improvement Measures

As mentioned previously, PHB presents with similar properties to some synthetic polymers including PE and PP in terms of their physical and mechanical properties. However, a number of differences and a reduced comparable thermal stability of PHB is evident. PHB materials are susceptible to thermal degradation when processed, and as a result a deterioration in the mechanical properties occurs [34]. The narrow process window is a primary factor responsible for this vulnerability to thermal degradation. In order to produce an overall, superior PHB material, a number of studies have been conducted aiming to improve the mechanical behavior and reduce the high costs associated with the material. Some of these approaches are based on chemical modifications of the material, through the addition of functional groups, obtaining copolymers and the use of blends.

It has been reported by Gopi et al. that the thermal properties of PHA materials can be altered via chemical modifications. This study reported that the Tg can be decreased by an increase in the length of the side chain, while simultaneously inducing a Tm increase [94]. In another report by Puppi et al., a similar trend was observed, where PHB without the presence of any additional alkyl side groups along the polymeric chain had a significantly lower Tg value when compared to standard PHB. It also showed a lower degree of crystallinity than standard PHB, decrease in stiffness and exhibiting a much larger elongation at break, resulting in improved processability of the material [6].

The chemical structure of PHB materials can also be chemically modified in order to enhance their properties. For example, genomic manipulation and plasmid construction aiming to alter PHB chemical structure has been reported recently. *Halomonas bluephagenesis*, a non-model halophilic bacteria serving as a framework for the Next Generation Industrial Biotechnology (NGIB), was successfully engineered to synthesize scl-co-mcl PHA copolymers such as poly-(3-hydroxybutyrate-co-3-hydroxyhexanoate) (P [3HB-co-3HH)]). The study summarized that modifying and optimizing the expression cassette and ribosomal binding site combined with the introduction of endogenous acetyl-CoA synthetase (fadD), resulted in a surprisingly high functional 3-hydroxyhexanoate (3HHx) monomer ratio when grown on glucose and 5-hexenoic acid as co-substrates. The resulting functional PHA material was shown to have good thermal stability and more than 1000% elongation at break [11]. 

In addition, functional groups can be added into the PHA side chain, such as double or triple bonds, methyl, epoxy, hydroxyl, carbonyl, cyano, phenyl, benzoyl, and halogen atoms, which can alter the chemical structure and properties of the resulting material. The introduction of functional groups allows for chemical modifications, which can dramatically expand the diversity of PHAs and tailor their physical features and properties. Depending on the functional groups introduced into the PHA side chain, changes of the pH, temperature, and moisture, as well as providing changes to many mechanical and physical properties can be induced [12,13]. In the recent study completed by Bhatia et al., PHBV was functionalized with ascorbic acid resulting in a lower degree of crystallinity (9.96%), a higher thermal degradation temperature and hydrophilicity when compared to the un-functionalized PHBV copolymer [15]. Another approach to prepare the PHA polyester block copolymers is to link PHAs and telechelic polyester oligomers with a coupling agent, such as diisocyanate and terephthaloyl chloride. Initial results have indicated that the thermal and mechanical properties of the PHB/PCL poly(ester urethanes) can be successfully manipulated by changing the block lengths of the hard and soft segments [95].

Producing blends with PHA polymers is currently being evaluated as an efficient and promising route to broadening the applications of PHA materials, taking advantage of fine tuning the functional properties by adding and adjusting the component make up. Additionally, PHB blends may also allow for the production of less expensive bioplastic materials, while improving the mechanical properties of the material. PLA is one material that is being evaluated to make a blend with PHB as it has several attractive properties such as its biodegradability, good mechanical properties, and is readily processible. There are several approaches concerning enhancing compatibility and miscibility between these materials. Some strategies include reactive melt-blending as an inducer of chemical reactions between polymers’ chains or introducing compatibilizers with the ability to modify morphology and properties of the mixture [96]. Zembouai et al. studied the blend properties of PHBV and PLA through melt mixing. In the blends produced, the crystalline index was found to decrease by 25, 44, and 83% as the weight ratio of PLA increased to 25, 50, and 75, respectively. The authors also reported that the Td, for all the blends evaluated was between that of PHBV and PLA and the thermal stability of the blends could be enhanced by increasing the percentage of PLA [14]. In the other hand, it has been reported that, using clays fillers, compatibility between these two polymers and thermal stability of resulting blends can be enhanced [97].

## 4. Conclusions

Billions of tones of plastic materials derived from non-renewable resources have been accumulated over the last 70 years now, with more than 85% reaching and remaining in the environment. In the last few decades, awareness of the destructive impact plastics have on nature has prompted developments in new disposal methods (sorting, recycling, composting, incineration) and laws against single-use plastics. It is becoming widely recognized that biopolymer developments are central to the solution for plastics circularity and intensified research efforts on renewable and regeneratable bioplastics which is now underway.

PHAs are a group of biopolymers showing significant potential to overcome and surmount petrochemical plastics sustainability and circularity issues. Among this group, PHB is the most examined and commonly used material, given its high potential mechanical performance properties. PHB can be obtained by microbial fermentation. This biopolymer is biocompatible and biodegradable and potentially an ideal alternative to synthetic polymers for applications in environmental, packaging, veterinary and medical market sectors.

This paper reviews fermentation process approaches employed for PHB production to date, their effects on resulting material properties and the possibilities of their improvement and optimization. It was found that discontinuous processes, which are well known and established methods for PHB materials production, provide limited productivity and non-consistent product quality, even with the adoption of different feeding strategies. Continuous processes in the other hand, are considered to be the simplest and the most ideal method for the PHB production. This type of process provides not only controlled conditions and high productivity, but also uniform product quality and a decrease in investment costs. Although continuous processes are more efficient in the case of PHB production, these processes are in general prone to contamination which can lead to a financial losses. In addition to choice of process, a number of factors are identified that can have an impact on the thermal and mechanical properties of the PHB material such as: choice of bacterial strain, carbon source, fermentation apparatus, agitation parameters, the recovery method and secondary crystallization factors. The final molecular weights and degree of crystallinity of resulting PHBs are dependent on the type of bacterial producing strain. Use of genetic engineering techniques and the development of recombinant strains are important ways to improve product quality. Proper choice of carbon source and cultivation conditions can lead to improved final product properties, as well as minimizing capital and operating costs such as substrate price and energy consumption. Cost effective substrates, such as waste streams from different industries present as ideal potential sustainable solutions, however only in case where the final product satisfies quality requirements. Type of used solvent and conditions in downstream processing can have significant effects on elasticity and thermal properties of the recovered PHB. Altering PHB characteristics can also be performed using various chemical and/or mechanical methods such as blending with other polymers, obtaining copolymers, the addition of functional groups, insertion of additives (plasticizers, nucleating agents, or photostabilizers). The preparation of blends, through processing and compounding is currently a quick and more attainable route to achieve desired properties of material relative to copolymer production. Future developments present a promising set of technologies to achieve PHBs with properties equating with and surpassing corresponding petroleum counterparts while fulfilling circularity requirements.

While PHB can be derived from renewable sources, current high production costs is the most important drawback to the mainstream application of this material. As the PHB production market grows, many more new and emerging fermentation processes will be implemented within industrial settings. The factors influencing the chemical and mechanical properties of the resulting material described here will be assessed and optimized. Continual improved bio-engineering processes have the potential to produce efficient high yield high performance PHBs, even from waste stream resources driving down costs and delivering price competitive PHB materials with the prerequisite mechanical properties to open up a broad span of new applications and market opportunities.

## Figures and Tables

**Table 1:** Summary of mechanical properties of P3(HB) and petrochemical based (PP, PET, PE) and bio-based polymers (PLA).

Mechanical Property	P3HB	PP	PET	LDPE	HDPE	PLLA	PDLLA
Tensile modulus (GPa)	3–3.5	1.95	9.35	0.26–0.5	0.5–1.1	2.7–4.14	1–3.45
Tensile Strength (MPa)	20–40	31–45	62	30	30–40	15.5–150	27.6–50
Elongation at break (%)	5–10	50–145	230	200–600	500–700	20–30	1.5–20
Degree of Crystallinity (%)	50–60	42.6–58.1	7.97	25–50	60–80	13.94	3.5
Melting Temperature (°C)	165–175	160–169.1	260	115	135	170–200	amorphous
Glass Transition Temperature (°C)	5–9	−20–−5	67–81	−130–100	−130–100	50–60	50–60

**Table 2:** Comparison of PHB materials’ thermal properties produced by different bacterial strains with literature values (Xc—degree of crystallinity, Tm—melting temperature, Tg—glass transition temperature).

Mechanical Property	Literature Values	PHB from *Bacillus megaterium*	PHB from *C. nector*
Xc (%)	53.4	23–37	46–53
Tm (°C)	169	151–176	169–175
Tg (°C)	1.1	−1–4	−0.2–0.6

**Table 3:** Summary of the results obtained when different fermentation mediums were used for PHB production (Xc—degree of crystallinity, Tm—melting temperature, Tg—glass transition temperature) [73,74].

Carbon Source	Fermentation Medium	Tg (°C)	Tm (°C)	Xc (%)
Soy Cake	Batch SSF in non- supplemented medium	−0.3	170.4	46
Soy Cake	Batch SSF in supplemented medium	−0.2	169.5	45
Soy Cake	Batch Submerged	1.1	173	53
Glucose/Fructose	Batch Submerged (0% oleic acid)	−4	173	70
Glucose/Fructose	Batch Submerged (0.9% oleic acid)	0	172	62
Glucose/Fructose	Batch Submerged (3.0% oleic acid)	−10	149	53

**Table 4:** The effects of the substrate cost and PHB yield used on PHB production [81].

Substrate	Substrate Cost (US $/kg)	PHB Yield	Production Cost (US $/kg)
Glucose	0.493	0.38	1.3
Sucrose	0.29	0.4	0.72
Methanol	0.18	0.43	0.42
Acetic acid	0.595	0.38	1.56
Ethanol	0.502	0.5	1
Cane molasses (waste-based substrate)	0.22	0.42	0.52
Cheese whey (waste-based substrate)	0.071	0.33	0.22
